# Risk of falls in hospitalized pregnant women: a descriptive, correlational study

**DOI:** 10.1590/1806-9282.20252205

**Published:** 2026-08-03

**Authors:** Sibel Kiyak, Hilal Türkben Polat

**Affiliations:** 1 Necmettin Erbakan University, Seydişehir Kamil Akkanat Faculty of Health Sciences, Department of Obstetrics and Gynecology Nursing – Konya, Türkiye.; 2 Necmettin Erbakan University, Seydişehir Kamil Akkanat Faculty of Health Sciences, Department of Fundamentals of Nursing – Konya, Türkiye.

**Keywords:** Pregnant women, Fall, Obstetrics, Risk

## Abstract

**OBJECTIVE:**

The aim of this study was to examine the risk of falls and associated factors among hospitalized pregnant women and to evaluate the measurement consistency between the Itaki II Fall Risk Scale and the Assessment Scale for Risk of Falling in Pregnant Women.

**METHODS:**

This descriptive, correlational study was performed in the obstetrics ward of a university hospital between March and August 2024. A total of 165 pregnant women were included in the study. Data were collected using the Assessment Scale for Risk of Falling in Pregnant Women questionnaire and the Itaki II Fall Risk Scale. For data analysis, the Mann-Whitney U test, Kruskal-Wallis H test, Spearman's correlation, linear regression, and Bland-Altman analysis were employed.

**RESULTS:**

The prevalence of falls during pregnancy was 10.3%. The median total Assessment Scale for Risk of Falling in Pregnant Women score was 9, whereas the median Itaki II Fall Risk Scale score was 2. Gestational weight gain, gestational age, spouse's education level, and the presence of chronic diseases were significantly associated with fall risk (p<0.05). A low-level significant correlation was found between the scales, and Bland-Altman analyses indicated that there was no measurement coherence between the two scales.

**CONCLUSION:**

Early identification of pregnant women at risk of falls and implementation of appropriate preventive strategies are critical for patient safety. Therefore, it is recommended to develop a reliable fall risk assessment tool tailored for hospitalized pregnant women and to provide education programs and environmental modifications.

## INTRODUCTION

Pregnancy is a period during which various hormonal, anatomical, and physiological changes occur within the body. During pregnancy, pregnant women experience several changes, including increased body weight, alterations in balance, loosening of connective tissues, and expansion of the abdominal area^1^. These changes can increase the risk of falls during pregnancy. Falls during pregnancy are among the most common causes of maternal and fetal injuries^2^. Pregnant women are not often considered a high-risk group for falls; however, falls are the second most common reason for emergency department visits among pregnant women. In the literature, fall rates during pregnancy were reported to be between 12 and 25%^3,4^. Falls during pregnancy can lead to severe obstetric complications, such as fractures, joint sprains, head trauma, internal bleeding, placental abruption, and preterm labor^5,6^. These complications can threaten the health of both the mother and the fetus and, therefore, make the assessment and prevention of fall risk during pregnancy critically important.

In case of pre-pregnancy health issues, pregnancy-related medical complications, obstetric problems, or fetal issues, hospitalization may be necessary for pregnant women to receive appropriate care and treatment^7^. Identifying the risk of falls in hospitalized pregnant women is an important component of maternal health because falls during pregnancy can cause significant health problems for both the mother and the fetus. Physiological changes during pregnancy, such as changes in balance, reduced muscle strength, and shifts in the center of gravity, when combined with environmental factors like poor lighting and cluttered spaces, increase the risk of falls in this vulnerable population^8^. Moreover, some medications prescribed during pregnancy can further increase the risk of falls^9^ and necessitate meticulous monitoring and risk assessment in hospital settings.

The Itaki Fall Risk Scale was used to identify the risk of falls among pregnant women at maternity clinics in Türkiye. This tool can also be used for elderly individuals, individuals with chronic illnesses, and patients in intensive care^10^. Although fall rates were reported to be similar between pregnant women and elderly individuals in the literature, there are differences in the causes and risk factors. When examining the causes of falls among pregnant women, pregnancy-related factors are typically predominant^11^. Not using fall risk assessment tools tailored for pregnant women can complicate the assessment process and hinder the implementation of appropriate preventive measures. Assessing fall risk among pregnant women is critically important for nurses to ensure continuity of care. These assessments enable nurses to implement targeted preventive measures, such as environmental modifications and individualized care plans^12^. Thus, patient safety and care quality can be improved, significantly reducing the risk of falls. The aim of this study is to examine the risk of falls and associated factors among hospitalized pregnant women and to evaluate the measurement consistency between the Itaki II Fall Risk Scale and the Assessment Scale for Risk of Falling in Pregnant Women (PASRoF).

## METHODS

### Design setting and study population

This descriptive, correlational study was conducted in a university hospital located in the Central Anatolia region of Turkey between March and August 2024. The hospital was selected due to its high patient volume and its service to patients with diverse sociodemographic characteristics. The study population consisted of pregnant women hospitalized in the obstetrics ward during the study period. The sample size was calculated using G*Power version 3.1.9.4. The power analysis was based on a linear regression model. The significance level (α) was set at 0.05, the statistical power (1-β) at 0.85, and the effect size was defined as f^2^=0.056^13^. According to the F-test, the required minimum sample size was determined to be 163 participants. A total of 170 participants were assessed for eligibility; 2 were excluded for being under 18 years of age and 3 for not being literate in Turkish. The study was completed with 165 pregnant women. Pregnant women hospitalized in the obstetrics ward who met the inclusion criteria were consecutively recruited using a convenience sampling method. Data were collected by the researchers through face-to-face interviews in the patient rooms. Completion of the questionnaires took approximately 15 min. The study involved pregnant women aged 18 years and older, who were literate in Turkish, and had no communication barriers. Pregnant women with a diagnosed neurological or psychiatric disorder, those with mobility limitations, or those requiring continuous assistance for ambulation were excluded from the study. These exclusion criteria were established to prevent confounding effects on fall risk. To minimize selection bias, participants were included in the study based on predefined inclusion and exclusion criteria. To reduce information bias, validated and reliable measurement instruments were used. In addition, simple and multiple regression analyses were performed to examine the relationships between variables and to assess their independent effects.

### Measurement

#### Questionnaire form

The questionnaire, developed by the researcher by reviewing the literature, included 25 questions assessing the participants’ sociodemographic, obstetric, and fall-related features.

#### Assessment Scale for Risk of Falling in Pregnant Women

Developed by Koç and Şahin^11^, this scale comprises 42 items scored as Yes: 1 or No: 0, based on the presence of risk factors for falls in pregnant women. The total scale score ranges from 0 to 42, and higher scores indicate an increased risk of falls in pregnant women. The Cronbach's alpha value of this scale was 0.604 in the original study^11^ and was calculated as 0.560 in this study.

#### Itaki II Fall Risk Scale

The Itaki Fall Risk Scale has been used in all public hospitals in Türkiye since 2015 as part of health quality requirements. Developed by the Ministry of Health, the scale has been revised and updated to the Itaki II Fall Risk Scale. The threshold value indicating high risk was updated to 10 points or higher, with patients scoring 10 points or more considered at high risk of falls^10^. Despite the low Cronbach's alpha coefficient of 0.46 reported in the literature^14^, this scale was used in the present study for its common application at the hospital where this study was conducted and for its recommendation by the Ministry of Health. In this study, Cronbach's alpha was 0.227.

#### Variables

The dependent variable in this study was the total PASRoF score. Independent variables included age, gestational week, pre-pregnancy body mass index (BMI), gestational weight gain, chronic disease, parity, and spouse's education level. BMI was calculated as body weight in kilograms divided by the square of height in meters (kg/m^2^). Gravidity was categorized as primigravida and multigravida. The spouse's education level was categorized as elementary school, high school, university, and above, and included in the model using dummy variables.

### Ethical approval

Ethical approval (Date 2024, No. 691), institutional permission (Date 2024, No. 486315), and informed consent from the participants were obtained before the study. The study was conducted in accordance with the principles of the Declaration of Helsinki.

### Data analysis

The analysis was conducted using IBM SPSS V27. Normality was tested using the Kolmogorov-Smirnov test. There were no missing data or losses during the study, and all participants were included in the final analysis. Group comparisons of total scores from the PASRoF were performed by utilizing the Mann-Whitney U test and the Kruskal-Wallis H test. Post hoc analysis of differences between groups was conducted with the Dunn test. Relationships between continuous variables were examined using Spearman's correlation coefficient, and the relationship between the two scales was evaluated by making use of the interclass correlation coefficient. Agreement between the scales was analyzed using the Bland-Altman method. Simple and multiple linear regression analyses were performed to examine the relationships between variables. While simple analyses evaluate crude associations, multiple regression analysis was used to assess the independent effects of variables^15^. Results were presented as mean±standard deviation and median (minimum–maximum). Statistical significance was set at p<0.05.

## RESULTS

A total of 165 pregnant women were included in the study. In this study, the mean age of pregnant women was 28.18±6.14 years. Among the respondents, 35.2% completed elementary education and 32.1% had chronic illness. Of the pregnant women, 41.2% were experiencing their first pregnancy and 62.4% had a planned pregnancy (Table 1). The mean gestational age was 25.05±9.60 weeks. The average length of hospital stay was 2.73±1.58 days.

**Table 1:** Comparison of descriptive characteristics of pregnant women with the total score of the fall risk assessment scale in pregnant women.

Variables		n (%)	PASRoF	Test value	p-value
Education					
	Elementary school	58 (35.2)	9 (3–17)		
	High school	57 (34.5)	9 (3–17)	2.235†	0.327
	University and above	50 (30.3)	8.5 (3–23)		
Working status					
	No	140 (84.8)	9 (3–23)	1,880‡	0.553
	Yes	25 (15.2)	10 (5–16)		
Education of the spouse					
	Elementary school	65 (39.4)	11 (3–17)^b^	10.878†	**0.004**
	High school	52 (31.5)	9 (4–23)^a^		
	University and above	48 (29.1)	8 (3–16)^a^		
Income status					
	Income less than expenses	7 (4.2)	8 (3–15)	0.10†	0.947
	Income equal to expenses	104 (63)	9 (3–23)		
	Income more than expenses	54 (32.7)	9 (3–17)		
Chronic illness					
	No	112 (67.9)	8 (3–17)	4,011‡	**<0.001**
	Yes	53 (32.1)	11 (5–23)		
Smoking					
	No	150 (90.9)	9 (3–23)	1,380.5‡	0.146
	Yes	15 (9.1)	11 (6–16)		
Gravidity					
	Primigravida	68 (41.2)	9 (3–23)	3,728.5‡	0.152
	Multigravida	97 (58.8)	8 (3–17)		
Number of living children					
	0	86 (52.1)	9 (3–23)		
	1	39 (23.6)	9 (3–15)	3.930†	0.269
	2	25 (15.2)	11 (5–15)		
	3 and above	15 (9.1)	10 (5–17)		
Type of pregnancy					
	Planned	103 (62.4)	9 (3–23)		
	Unplanned	49 (29.7)	9 (3–17)	0.351†	0.839
	With treatment	13 (7.9)	9 (4–14)		
Continuous medication use during pregnancy					
	No	77 (46.7)	9 (3–17)	3,127‡	0.392
	Yes	88 (53.3)	9 (3–23)		

† Kruskal-Wallis H test

‡ Mann-Whitney U test.

a,b There is no difference between groups with the same letter for each measurement (Dunn test). PASRoF: Assessment Scale for Risk of Falling in Pregnant Women. Bold values indicate statistical significance (p<0.05).

A total of 17 women (10.3%) experienced at least one fall during pregnancy. The average time after the fall was 60.62±58.65 days. Of these falls, 70.6% occurred at home, 17.6% on the street, 5.9% at the workplace, and 5.9% at the hospital. Among those who fell, 29.4% fell while walking on a slippery surface, 23.5% fell when getting up from bed at night, and 17.6% fell due to fainting. After the fall, 29.4% of the pregnant women sustained injuries. Among those injured, 40% experienced sprains, 40% bruising, and 20% reported back pain.

In this study, the median value of the PASRoF was 9 (min: 3–max: 23) and the median value of the Itaki II Fall Risk Scale was 2 (min: 0–max: 14). It was determined that 0.6% of pregnant women had a risk of falling (Itaki>10).

In this study, pregnant women whose spouses had an elementary school and those with a chronic illness were found to have higher fall risk scores (p=0.004 and p<0.001, respectively). However, no significant difference was found in the mean total score of the PASRoF in terms of obstetric characteristics (p>0.05) (Table 1).

Considering the results obtained from correlation analysis, there was a positive and significant correlation between the total score of the PASRoF and gestational age (r=0.277, p<0.001). The intraclass correlation coefficient showed a low but significant relationship between the PASRoF and the Itaki II Fall Risk Scale (ICC=0.371; 95%CI 0.232:0.495; p<0.001).

Table 2 presents the results of simple and multiple linear regression analyses examining the variables associated with the total PASRoF score. The regression model showed that gestational age, gestational weight gain, presence of chronic disease, and spouse's education level were significantly associated with the PASRoF total score (p<0.05) (Table 2).

**Table 2:** Linear regression analysis results for the Assessment Scale for Risk of Falling in Pregnant Women total score.

Variables		Simple regression						Multiple regression					
		B (95%CI)	β	SE	T	p-value	B (95%CI)	β	SE	t	p-value
(Constant)							9.251 (5.978–12.523)	0	1.657	5.584	
Age (years)		-0.042 (-0.129–0.044)	-0.076	0.044	-1.000	0.335	-0.084 (-0.178–0.009)	-0.151	0.047	-1.784	0.076
Gestational age (weeks)		0.079 (0.025–0.133)	0.221	0.027	2.894	**0.004**	0.111 (0.053–0.17)	0.310	0.030	3.751	**<0.001**
Pre-pregnancy BMI (kg/m_2_)		0.049 (-0.055–0.154)	0.073	0.053	0.932	0.353	0.005 (-0.096–0.105)	0.007	0.051	0.095	0.925
Weight gained during pregnancy (kg)		0.001 (-0.080–0.081)	0.002	0.041	0.020	0.984	-0.091 (-0.177 to −0.004)	-0.174	0.044	-2.078	**0.039**
Chronic illness		2.229 (1.147–3.311)	0.304	0.548	4.069	**<0.001**	2.326 (1.299–3.353)	0.317	0.520	4.475	**<0.001**
Gravidity		0.582 (-0.491–1.655)	0.084	0.544	1.071	0.286	0.801 (-0.351–1.953)	0.115	0.583	1.373	0.172
Education of the spouse											
	High school	-1.235 (-2.471–0.001)	-0.167	0.626	-2.000	**0.050**	-1.341 (-2.512 to −0.17)	-0.182	0.593	-2.262	**0.025**
	University and above	-1.890 (-3.154 to −0.626)	-0.250	0.64	-2.952	**0.004**	-1.741 (-2.953 to −0.53)	-0.231	0.613	-2.839	**0.005**

F=6.079, p<0.001, Adj.R^2^=0.199, R^2^=0.238, SE=3.077, Durbin-Watson=1.936, The highest VIF and tolerance: 1.459 and 0.975. Note: p<0.05 was considered statistically significant. **Β**: unstandardized beta β: standardized beta; SE: standard error; CI: confidence interval; BMI: body mass index. Bold values indicate statistical significance (p<0.05).

**Figure 1:**
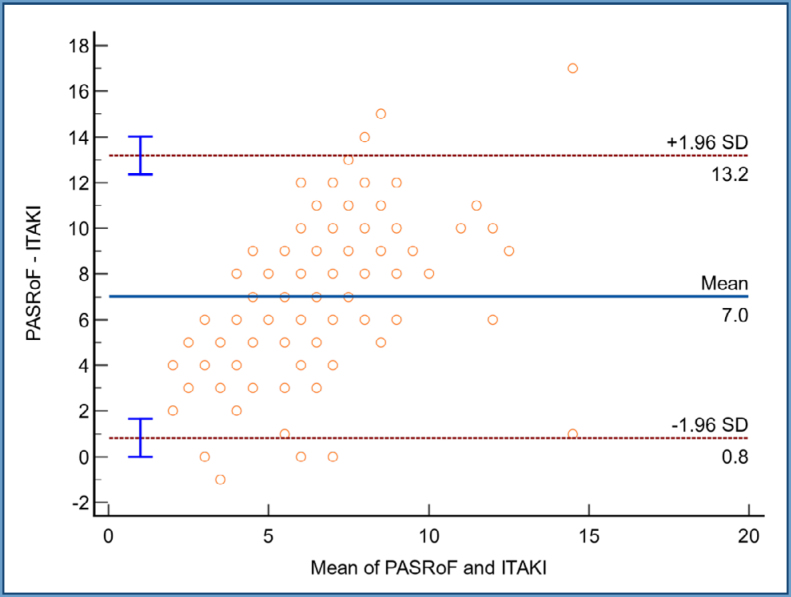
Bland-Altman analysis results.

The results of the Bland-Altman analysis between the PASRoF and Itaki II Fall Risk Scale are presented in Figure 1. In this analysis, the difference between the two scales is shown on the Y-axis, whereas the mean values obtained from both scales are shown on the X-axis. The proportional bias between the two scales was found to be significant (t=7.006; 95%CI 6.521:7.490; p<0.001). These results indicate that the two scales do not provide compatible measurements.

## DISCUSSION

The aim of this study is to examine the risk of falls and associated factors among hospitalized pregnant women and to evaluate the measurement consistency between the Itaki II Fall Risk Scale and the Assessment Scale for Risk of Falling in Pregnant Women (PASRoF). The results obtained here indicate that the risk of falling among hospitalized pregnant women is low; however, factors such as gestational weight gain, gestational age, spouse's education level, and the presence of chronic diseases were significantly associated with fall risk. A low-level significant correlation was found between the scales, and Bland-Altman analyses indicated that there was no measurement coherence between the two scales.

Fall risk during pregnancy is a significant concern because it can lead to potentially serious complications for both the mother and the fetus. The history of falls is recognized as an important risk factor and is therefore included in many falls risk assessment tools^12^. According to previous studies, the frequency of falls during pregnancy ranges from 12 to 27%^3,16^. In our study, the frequency of falls among pregnant women was 10.3%. Factors such as sample size, demographic differences, and cultural factors may affect the frequency of falls.

Most injuries reported during pregnancy result from falls occurring during daily activities^12^. Slippery floors, cluttered and uneven surfaces, unsuitable footwear, hurried movements, inadequate lighting, and a sedentary lifestyle can all increase the risk of falling during pregnancy^8^. In the present study, the causes of falls were walking on slippery surfaces (29.4%), getting out of bed at night (23.5%), and fainting (17.6%). Pregnant women reported stairs (39.8%), slippery floors (35.2%), and rushing (35.2%) as the main reasons for falls during pregnancy^17^. Similarly, another study found that the causes of falls among pregnant women were reported to be climbing or descending stairs (16.8%), wet floors (16.8%), and sloped/uneven surfaces (13.9%)^18^. These results support the impact of environmental and behavioral risk factors on fall incidents and highlight the need for educating and raising awareness among pregnant women about these risks. In this study, 29.4% of the pregnant women, who fell, experienced injuries. Among injured pregnant women, 40% reported sprains, 40% bruising, and 20% lower back pain. It has been indicated in the literature that the injury rate after falls among pregnant women ranges between 26.8 and 36.4%, with bruising, back pain, and sprains being the most common injuries^16,18^.

In this study, the median total score of PASRoF was 9. In comparison to the median value of 6 reported by Koç and Şahin^11^, this result suggests that the participants in the present study had a higher fall risk. This difference may be attributed to the sample characteristics. Specifically, the higher number of hospitalized and high-risk pregnancies in this study might have contributed to the higher risk scores. Furthermore, the hospital environment and length of hospitalization may negatively affect participants’ physical activity levels and mobility.

In this study, the median total PASRoF scores varied according to the spouse's education status. Individuals with lower education levels may have less awareness and knowledge about health and safety issues, which can lead to inadequacies in understanding and preventing fall risks.

It was also determined in this study that fall risk among pregnant women is associated with chronic diseases. Health conditions such as pre-existing diabetes, hypertension, and autoimmune disorders can complicate pregnancy and require more careful monitoring and intervention. These chronic diseases can affect balance and coordination, increasing the risk of falls among pregnant women. Moreover, hormonal and physiological changes during pregnancy can exacerbate the effects of chronic diseases^9^. Therefore, special attention and management are required to reduce fall risk in pregnant women with chronic diseases.

The risk of falling during pregnancy increases as pregnancy progresses. Consistent with the results obtained in the present study, fall risk increases with gestational age, particularly becoming more pronounced in the third trimester^1,3,8,18^. This can be explained by factors such as weight gain during pregnancy, changes in the center of gravity, and joint relaxation due to hormone relaxation, which increase the risk of falls. In addition, enlargement of the abdomen and reduced mobility are other significant factors that increase the risk of this condition^1,19^.

Previous studies have shown that greater gestational weight gain is associated with an increased risk of falls during pregnancy^13,20^. However, in this study, an inverse relationship was observed, with gestational weight gain being associated with a reduced risk of falls. This difference may be explained by reduced physical activity and more cautious movement among women with higher weight gain.

In Türkiye, the Itaki Fall Risk Scale is used to assess fall risk in obstetric clinics and other patient groups (e.g., the elderly, those with chronic diseases, and intensive care patients). However, the fall risk forms utilized in clinics may not fully capture the fall risk in pregnant women^11^. Therefore, the compatibility of two different fall risk scales in terms of their applicability to pregnant women was examined, and it was found that these scales are not compatible. This discrepancy could be explained by the fact that the physiological and psychological changes brought about by pregnancy differ from those observed in other patient groups and that the existing scales do not sufficiently account for pregnancy-specific risk factors.

This study has some limitations. First, the fact that the present study was conducted in a single tertiary hospital limits the generalizability of the findings to a broader population. Second, the measurement tools used in this study were developed and applied in Turkey; therefore, their cultural and contextual specificity may further restrict the generalizability of the results to other countries and populations. Third, retrospective identification of falls may introduce recall bias. Finally, the internal consistency of the Itaki Fall Risk II scale used in this study was low, which necessitated careful evaluation and interpretation of the results.

## CONCLUSION

This study demonstrates that a general clinical scale and a pregnancy-specific scale are not compatible in assessing fall risk during pregnancy, and this discrepancy may adversely affect clinical safety. Nurses and midwives working in obstetric units should evaluate fall risk not only as a physical condition but also as a multifactorial process influenced by gestational age, gestational weight gain, presence of chronic disease, and spouse's education level. In particular, in obstetric clinics in Türkiye, it is recommended that valid and reliable pregnancy-specific fall risk assessment tools be developed and integrated into routine antenatal care. Future research employing longitudinal designs to examine fall risk may contribute to the development of more comprehensive and effective safety strategies.

## Data Availability

The datasets generated and/or analyzed during the current study are available from the corresponding author upon reasonable request.
